# Influence of *SH2B3*, *MTHFD1L*, *GGCX*, and *ITGB3* Gene Polymorphisms on theVariability on Warfarin Dosage Requirements and Susceptibility to CVD in the Jordanian Population

**DOI:** 10.3390/jpm10030117

**Published:** 2020-09-09

**Authors:** Laith N. AL-Eitan, Ayah Y. Almasri, Rame H. Khasawneh, Mansour A. Alghamdi

**Affiliations:** 1 Department of Applied Biological Sciences, Jordan University of Science and Technology, Irbid 22110, Jordan; ayalmasri13@sci.just.edu.jo; 2 Department of Biotechnology and Genetic Engineering, Jordan University of Science and Technology, Irbid 22110, Jordan; 3 Department of Hematopathology, King Hussein Medical Center (KHMC), Jordan Royal Medical Services (RMS), Amman 11118, Jordan; rami.khasawneh@jaf.mil.jo; 4 Department of Anatomy, College of Medicine, King Khalid University, Abha 61421, Saudi Arabia; m.alghamdi@kku.edu.sa; 5 Genomics and Personalized Medicine Unit, College of Medicine, King Khalid University, Abha 61421, Saudi Arabia

**Keywords:** warfarin, warfarin dose, cardiovascular disease, pharmacogenetics, INR, *MTHFD1L*, *SH2B3*

## Abstract

The purpose of this study was to investigate the effects of the *SH2B3*, *MTHFD1L*, *GGCX*, and *ITGB3* gene variants on the efficacy of warfarin treatment and its effects on the risk of cardiovascular disorders in Jordanian patients. The selected genes and their polymorphisms are involved in many Genome-Wide Association Study (GWAS) associated with cardiovascular disease and the variability of warfarin therapy. The current study conducted a genetic association and pharmacogenetics study in (212) Jordanian cardiovascular patients treated with warfarin and (213) healthy controls. DNA extraction and the Mass ARRAY™ system were used to genotype ten selected polymorphisms within four genes (*SH2B3*, *MTHFD1L*, *GGCX*, and *ITGB3*). This study confirmed a genetic association of *MTHFD1L* rs6922269 Single Nucleotide Polymorphism (SNP) with warfarin sensitivity during the initial and stabilization phases of treatment. Moreover, this SNP showed significant differences in the initial and maintenance doses of warfarin. This study also found an association between the genetic haplotypes (AGC and GAT) within the *SH2B3* gene and responsiveness to warfarin. However, possession of an *MTHFD1L* rs491552 variant allele was found to affect the outcome measure of the international normalized ratio (INR) during the stabilization phase of warfarin treatment. In contrast, there was no association between all selected SNPs and susceptibility to cardiovascular disorders. This study extends the current understanding of the high variability of the warfarin response, including variability in dose requirements and susceptibility to cardiovascular disease in the Jordanian-Arab population. Other studies on a larger sample and in different ethnic groups could help to better understand the pharmacogenetics of warfarin and its application in personalized medicine.

## 1. Introduction

The term “pharmacogenetics” was developed in 1959 and defined as “the study of the variability of genome structure in response to drugs due to heredity”; since that time, numerous studies have been conducted to investigate the effects of genetic variants in the drug responses among individuals. However, warfarin is one of the best-known drugs for having a high variability in responses due to individual genetic variants [1,2]. Although it is the most commonly prescribed anticoagulant drug for the treatment of many cardiovascular diseases, including deep vein thrombosis, arterial thromboembolism, and heart valve replacement [3], the adjustment of warfarin doses has been complicated, due to its narrow therapeutic range, which was associated with bleeding or thrombosis in high or low doses, respectively [4]. Thus, regular monitoring of the anticoagulation status should be performed for each patient by measuring the international normalized ratio (INR) [5]. In addition to the environmental factors that include age, body mass index, smoking, gender, vitamin inducers, concomitant medication, and diet, genetic factors and ethnic characteristics affect the pharmacokinetics and pharmacodynamics of warfarin and lead to changes in the efficacy and safety between individuals [6]. Consequently, several studies on the pharmacogenetics of warfarin have been carried out, and it has been shown that the polymorphisms of some genes play an important role in predicting the warfarin dose. However, the most commonly studied genes were the *VKORC1* gene (encoding vitamin K epoxide reductase complex subunit 1), which is the target of warfarin, and the *CYP2C9* gene (cytochrome P450 superfamily) responsible for S-warfarin metabolism and clearance [7,8,9].

Recent studies have shown that polymorphisms in various genes are responsible for the variability of warfarin response and, therefore, have a significant effect on the warfarin required dose [10,11]. Among them, *GGCX* (gamma-glutamyl carboxylase), *CYP4F2* (cytochrome P450, family 4, subfamily F, polypeptide 2), *ApoE* (apolipoprotein E), and *FVII* (blood clotting factor VII) are genes that can help predict a stable therapeutic dose of warfarin [11,12,13,14,15]. Recently, several algorithms have been proposed to predict the appropriate warfarin dose for each patient by combining the *CYP2C9* and *VKORC1* variants with other clinical and environmental factors [16,17].In fact, there are many complications and challenges in the exchange of personal medicine into clinical settings, especially in the Arab population [18,19]. Therefore, it is necessary to expand studies on candidate genes involved in the pharmacokinetics and pharmacodynamics of warfarin to improve both the efficacy and safety of drugs. Examining several genes that affect warfarin metabolism and are involved in cardiovascular disease (CVD) may help to provide more accurate treatment by reducing the side effects [18,20].

For this reason, four genes (*SH2B3*, *MTHFD1L*, *GGCX*, and *ITGB3*) were analyzed to determine their influence on CVD risk, warfarin sensitivity, and responsiveness. The *SH2B3* gene belongs to the SH2B adaptors family. This gene is located on chromosome 12 (12k24) and encodes the *SH2B3* protein, which is responsible for the negative regulation of cytokine signaling [21]. However, it has been shown that genetic variability in this region is associated with multiple symptoms, including type one diabetes, risk of coronary heart disease, and myocardial infarction [22,23].

The methylene-tetrahydrofolate dehydrogenase (NADP + -dependent) 1-like gene (*MTHFD1L*) is involved in the mitochondrial metabolism of tetrahydrofolate [24]. However, the polymorphism of this gene is closely related to the development of coronary heart disease (CHD), as found in a recent meta-analysis [25]. Moreover, a family study reported that a significant association was found between *MTHFD1L* polymorphism and the risk of myocardial infarction (MI) [26]. The γ-glutamyl carboxylase (*GGCX*) gene encodes the main enzyme responsible for carboxylation of the glutamic acid residue in the vitamin K-dependent coagulation factor to obtain its activity. Thus, polymorphisms, including this gene, can affect the coagulation factor cascade, and, therefore, it can lead to variability on an individual’s susceptibility to stroke disease [27]. The *ITGB3* gene encodes the beta integrin subunit; this subunit, together with the alpha subunit encoding by *ITGA2B*, are involved in some of the specific platelet receptors for fibrinogen, a protein that regulates platelet aggregation, thrombosis, and hemostasis [28,29]. However, it was discovered that the *ITGB3* gene was a highly polymorphic region, and some of these polymorphisms were associated with cardiovascular diseases [30]. The objective of the present study was to demonstrate the impacts of *SH2B3*, *MTHFD1L*, *GGCX*, and *ITGB3* polymorphisms on the risk of CVD and the warfarin sensitivity and responsiveness in Jordanian patients.

## 2. Materials and Methods

### 2.1. Study Population

The study population included 425 subjects, of whom 212 were irrelevant warfarin outpatients recruited from the Anticoagulation Clinic at the Queen Alia Heart Institute (QAHI) in Amman, Jordan, and the remaining 213 were healthy noncardiovascular control groups. All participants accompanied the written consent of the Arab-Jordanian population. This study was approved by the Human Research Committees of the Jordan University of Science and Technology in Irbid and the Royal Medical Services of Amman. The enrolled patients met the following inclusion criteria: (1) patients who received warfarin for at least three months, (2) who undergo the anticoagulation clinic regularly, and (3) are older than 18 years. The most important exclusion criteria included: (1) patients without written consent or registered clinical data, (2) the use of *CYP2C9* inhibitors or induction drugs, (3) get concomitant medications that are interacting with warfarin, (4) alcohol consumption, and (5) pregnant women.

### 2.2. Data Collection and Follow-Up Time

Blood samples were taken to obtain the INR value and to genotype the selected SNPs. The medical records include the average weekly warfarin dose and INR values, indication for warfarin, bleeding events, and concomitant medication and demographic information (gender, smoking status, age, and body mass index) reported during the patient’s clinical visits through semi-standardized interviews and from patient’s medical records. Both warfarin doses and INR measurements were documented in two stages. The primary stage is at the start of therapy, wherein the warfarin dose is determined dependent on the patient’s clinical factors and the indication of treatment, notwithstanding the genetics factor. Therefore, the INR measurement is unstable and is referred to as the initiation phase of treatment [31,32]. In the second stage, where the INR measurement is stabilized within the therapeutic range for at least three consecutive clinical visits, this phase becomes referred to as the stabilization phase of treatment.

### 2.3. Outcome Measure

This study was split into two branches: genetic association study (comparison of selected SNPs between patients and controls) and pharmacogenetics study (exhibited the effects of selected SNPs on warfarin therapy during the initial and stabilization phases of treatment). However, the pharmacogenetics study was also divided into two basic categories. The sensitivity to warfarin was the first. Patients in this category were divided according to the warfarin required dose into warfarin-resistant patients (requiring higher doses to achieve a therapeutic INR (>49 mg/week)), normal patients (receiving moderate doses (21–49 mg/week)), and sensitive patients (require a lower dose (<21 mg/week)).The classes of this category were divided according to a study by Gordon et al. (2009) [33]. The second category involved responsiveness to warfarin and was based on Higashi et al. (2002), who divided patients into good responders (INR value within the therapeutic range), poor responders (INR below the target range), and ultra-responders (INR above the target range) [34].

### 2.4. SNP Selection and Genotyping

Ten SNPs of *SH2B3*, *MTHFD1L*, *GGCX*, and *ITGB3* genes were selected from a public database such as the National Center for Biotechnology Information (NCBI) (http://www.ncbi.nlm.nih.gov/SNP/) and the SNP of Applied Biosystems database (http://www.appliedbiosystems). The genes, SNP IDs, and their information locationsare listed in Table S1. The Wizard genomic DNA purification kit (Promega, Madison, WI, USA) was used to extract the DNA from the blood sample. Subsequently, samples that met the quantitative requirements were sent to the Australian Genome Research Center (AGRF) to test the genotype with the Mass ARRAYiPLEX GOLD system, Sequenom, San Diego, CA, USA. The Mass ARRAYTM system protocol and primer information are available upon request.

### 2.5. Statistical Analysis

To investigate which of the selected SNPs is associated with CVD risk and warfarin sensitivity and responsiveness, multivariate analyses, including a one-way ANOVA, chi-square test, Tukey’s honest significant differences (HSD) post hoc multiple comparison test, and nonparametric correlation tests, were performed. For all analyses, SPSS version 21.0 (SPSS Inc., Chicago, IL, USA) was used.

## 3. Results

### 3.1. Patient Characteristics

This study incorporated 212 cardiovascular patients treated with warfarin anticoagulants and 213 healthy controls recruited from Jordan. The genotypes of *SH2B3*, *MTHFD1L*, *GGCX*, and *ITGB3* polymorphisms, plus clinical data for the participants, were evaluated to investigate their effects on the risk of cardiovascular disease and warfarin sensitivity and responsiveness during the initial phase treatment. Of these, 139 patients (66%) achieved the stabilization phase; therefore, they were used to assess the genetic effects of the aforementioned polymorphisms on the response and sensitivity to warfarin during the stabilization phase of therapy. Overall, 69.6% of the patients were found to be good metabolizers, 15.2% were extensive metabolizers, and 15.2% poor metabolizers. The demographic and clinical characteristics of the patients in each group were confirmed by a previous study by AL-Eitan et al. (2019) [35].

### 3.2. Genotyping and Allelic Frequencies

Of the ten selected SNPs, three SNPs were not polymorphic (monomorphic): rs28928872 of the *GGCX* gene and rs398122372 and rs398122374 of the *ITGB3* gene; therefore, only seven SNPs were included in this study. The latter went beyond quality control, with high accuracy and a low discordance rate. The minor allele frequency and Hardy Weinberg equilibrium (HWE) *p*-values for all participants are summarized in Table S2. The genotypic frequency of the *SH2B3* and *MTHFD1L* genes in patients and controls are exhibited in Table 1.

For all selected SNPs, there was no statistically significant difference between patients and controls (*p* > 0.05). Whereas significant associations were found between *MTHFD1L* haplotypes (GGAG) and cardiovascular disease in patients and healthy controls (*p* = 0.04) (Table S3).

### 3.3. Association of SH2B3 and MTHFD1L Polymorphisms with Warfarin Sensitivity during the Initiation and Stabilization Phases of Therapy

Patients were characterized based on the dosage needed to reach therapeutic INR into three different groups: sensitive, moderate, and resistant groups. No significant association was observed between the *SH2B3* and *MTHFD1L* haplotypes and the warfarin sensitivity (*p* > 0.05) (Table S4). Moreover, all examined SNPs showed no significant differences between the warfarin groups and the sensitivity to warfarin during the initial and stabilization phases of therapy with *p* > 0.05, except for *MTHFD1L* rs6922269, which showed that 26.2% of the AG carriers were resistant to warfarin and, therefore, required higher doses to achieve the therapeutic INR, while only 5.9% of the wild-type AA was in the initial phase of the treatment with *p* = 0.03. Additionally, 36.9% of the rs6922269 AG carriers were resistant to warfarin and, therefore, required a higher dose to achieve the therapeutic INR, and only 18.2% of the wild-type AA were resistant in the stabilization phase of the treatment (*p* = 0.05) (Table 2, Table 3 and Table 4).

Furthermore, there were statistically significant differences between this SNP and the dosages required to reach the INR target during the initial and stabilization phases of treatment (*p* = 0.02 and *p* = 0.004, respectively) (Table 4). Post-hoc tests also showed significant correlations between this SNP and the required warfarin dose (*p*-value ≤ 0.05) (Table S5).

### 3.4. Association of SH2B3 and MTHFD1L Polymorphisms and Warfarin Responsiveness during Initiation and Stabilization Phases of Therapy

Depending on the response to warfarin, the patients were divided into three groups: poor, good, and ultra-responder. There were no significant differences between the frequencies of the different genotypes in the three groups in the initial phase and stabilization phase of the therapy with *p* > 0.05 (Table 5 and Table 6). However, a significant association was found with the *SH2B3* haplotypes AGC and GAT and the ability to respond to warfarin (*p* = 0.002) (Table S6). There were no significant differences between the selected SNPs and the INR values measured in the initial phase of therapy in 212 patients receiving warfarin (*p* > 0.05). However, differences were found between *MTHFD1L* rs491552 and the INR values measured during the stabilization phase in 139 patients with an overall *p*-value = 0.02 (Table 7 and Table S7).

### 3.5. Association between Warfarin Dose and Clinical Data

The correlation between the warfarin dose and clinical features was evaluated in previous studies using multi-regression analyses, and there was no significant association between the warfarin dose and age, body mass index, gender, treatment indication, or comorbidities (*p* > 0.5) [36].

## 4. Discussion

This study reports genotype polymorphisms for the *SH2B3*, *MTHFD1L*, *GGCX*, and *ITGB3* genes in association with the risk of CVD in Jordanian cardiovascular patients and the relationship between these polymorphisms in addition to non genetic factors for sensitivity and the responses to warfarin during the initial and stabilization phases of treatment. Our data suggest that there is an association between *MTHFD1L* polymorphism rs6922269 and warfarin sensitivity during the initiation and stabilization phases of treatment, and this indicates that this polymorphism plays an important genetic role for the variations in warfarin dose requirements. Moreover, our data revealed a significant association between *MTHFD1L* rs491552 polymorphism and the INR outcome measures during the stabilization phase of treatment. However, no correlation was found among all the polymorphisms studied and the risk of developing a cardiovascular disorder.

Our results regarding the genotype and allelic frequencies of the *GGCX* and *ITGB3* genes show that theses polymorphisms are not polymorphic (monomorphic), which means polymorphisms in these two genes are not found in our population; therefore, they were excluded from our analysis. While the minor allele frequencies for rs11065987, rs17696736, and rs3184504 SNPs within the *SH2B3* gene in our population are 38%, 38%, and 39%, respectively, which is inconsistent with the frequencies in the European population, with 34%, 42%, and 40–49%, respectively [37,38,39]. For the polymorphisms rs6922269 and rs803422 within the *MTHFD1L* gene, the minor allele frequency in our population for both is 26%, which is in agreement with the European population with 27% [24]. While the rs491552 SNP is high in our population, with 46%, the rs803455 is low, with only 9%. Although several previous studies have shown a significant association between rs11065987, rs17696736, and rs3184504 SNPs within the *SH2B3* gene and susceptibility to cardiovascular diseases, including blood pressure, ischemic stroke, myocardial infarction, and coronary artery disease, our results failed to find this correlation in our population (*p* > 0.05) (Table 1) [23,40,41]. For *MTHFD1L* SNPs, our results showed no association between these SNPs and CVD risk (*p* > 0.05) (Table 1),while rs6922269 was observed to be significantly associated in the previous study with the risk of CVD, and that was first reported by a meta-analysis of two large genome-wide association studies [26]. Inaccordance with our results, other studies showed no significant association between this polymorphism and the risk of CVD [25,42].

To our knowledge, no studies have investigated the correlation between *SH2B3* and *MTHFD1L* polymorphisms and the effectiveness of warfarin treatments. In this study, we examined the relationship between these polymorphisms and the sensitivity and responsiveness of warfarin therapy in the initial and stabilization phases of therapy in Jordanian cardiovascular patients. The results of this pharmacogenetics study suggest that, in both treatment phases, there was no significant association of *SH2B3* polymorphisms with the sensitivity and responsiveness of warfarin (*p* > 0.05) (Table 2, Table 3, Table 4, Table 5 and Table 6), except for a significant relationship between *SH2B3* haplotypes and the response to warfarin (*p* = 0.002) (Table S6). Meanwhile, our results showed that (rs6922269) was associated with warfarin sensitivity during the initiation phase of the treatment.

We found that patients with the variant allele A are at an increased risk of warfarin resistance. With 26.2% of the patients with AG, they were resistant to warfarin, while only 10.4% of the homozygous of the wild-type allele GG were resistant (overall, *p* = 0.003) (Table 2). Consequently, our results showed that patients with AG required 43.91 mg/week, while homozygous GG patients required only 34.49 mg/week (*p* = 0.02) (Table 4). Besides, this SNP tends towards statistical significance in relation with sensitivity to warfarin during the stabilization phase of the treatment, in which it was found that 36.8% of patients with AG were resistant to warfarin, and only 14.1% of GG was resistant (overall, *p* = 0.05) (Table 3). In accordance, our results showed that patient carriers for AG required 44.52 mg/week, while patients homozygous for GG required 34.28 mg/week only (*p* = 0.00) (Table 4).

Regarding warfarin responsiveness, our results did not show a significant association between the selected SNPs and the ability to respond to warfarin during the initiation phase. Although the rs491552 MTHFD1L SNP did not show a statistically significant value, it was close to being significant (*p* = 0.07) (Table 5). The patients carrying the variant allele G showed a high risk of being ultra-responders (12.1%), so the INR value over the target INR (their average INR values were 2.61), while 0.0% of the wild-type allele AA carriers were ultra-responders, and their INR values were 2.3 (*p* = 0.03) (Table 7).

## 5. Conclusions

Our data show the lack of a significant association between the *SH2B3* and *MTHFD1L* SNPs and CVD risk in the Jordanian population. On the other hand, the *MTHFD1L* rs6922269 SNP showed a significant association with sensitivity and response to warfarin during the initial and the stabilization phases of the treatment. However, there was a significant relationship between this SNP, and the variability of warfarin required doses during both therapy phases. A significant association was also found between the *MTHFD1L* rs491552 SNP and the ability to respond to warfarin and the INR outcome values. To verify our results, more research is needed on larger sample sizes and on different ethnic populations. Individualized warfarin therapy should be done to maintain a safe and effective anticoagulation treatment in patients with cardiovascular disease. Thus, further pharmacogenetics studies are needed to evaluate the effects of other clinical and genetic factors and to facilitate the prevention and treatment of cardiovascular diseases.

## Figures and Tables

**Table 1:** The distributions of *SH2B3* and *MTHFD1L* SNPs in 212 cardiovascular patients and 213 healthy controls.

Gene	SNP ID	Model	Patients %	Controls %	*p*-Value *
*SH2B3*		AA/GA/GG	39.6/42.9/17.4	42.2/41.3/16.4	0.86
	rs11065987	AA/(GA + GG)	39.6/60.4	42.2/57.8	0.58
		(AA + GA)/GG	82.5/17.4	83.6/16.4	0.78
		AA/AG/GG	39.5/42.4/18.1	42.5/42.5/15.1	0.67
	rs17696736	AA/(AG + GG)	39.5/60.5	42.5/57.5	0.54
		(AA + AG)/GG	81.9/18.1	84.9/15.1	0.41
		CC/TC/TT	38.7/42/19.3	41.8/42.2/16	0.62
	rs3184504	CC/(TC + TT)	38.7/61.3	41.8/58.2	0.51
		(CC + TC)/TT	80.7/19.3	84/16	0.36
*MTHFD1L*		GG/CG/CC	28.4/49.5/22.1	33.5/41.3/25.2	0.24
	rs491552	GG/(CG + CC)	28.4/71.6	33.5/66.5	0.27
		(GG + CG)/CC	77.9/22.1	84.8/25.2	0.45
		GG/AG/AA	54.2/37.7/8	55.9/37.6/6.6	0.84
	rs6922269	GG/(AG + AA)	54.2/45.8	55.9/44.1	0.74
		(GG + AG)/AA	92/8	93.4/6.6	0.57
		AA/GA/GG	53.3/35.4/11.3	59.6/34.3/6.1	0.12
	rs803422	AA/(GA + GG)	53.3/46.7	59.6/40.4	0.19
		(AA + GA)/GG	88.7/11.3	93.9/6.1	0.06
		AA/GA/GG	86.7/12.8/0.5	79.8/18.3/1.9	0.1
	rs803455	AA/(GA + GG)	86.7/13.3	79.8/20.2	0.06
		(AA + GA)/GG	99.5/0.5	98.1/1.9	0.17

* Chi–square test with *p *< 0.05 is considered significant.

**Table 2:** Association of *SH2B3* and *MTHFD1L* SNPs with warfarin sensitivity during the initiation phase of therapy of 212 cardiovascular patients treated with warfarin.

Gene	SNP ID	Genotype	Sensitive ^a^	Moderate ^b^	Resistance ^c^	Overall *p*-Value *
*SH2B3*	rs11065987	AA	(14/84) 16.7%	(57/84) 67.9%	(13/84) 15.5%	0.99
		*p*-value *	0.87	0.97	0.99	
		GA	(13/91) 14.3%	(63/91) 69.2%	(15/91) 16.5%	
		*p*-value *	0.96	1	0.99	
		GG	(5/37) 13.5%	(26/37) 70.3%	(6/37) 16.2%	
		*p*-value *	0.96	0.98	1	
	rs17696736	AA	(13/83) 15.7%	(57/83) 68.7%	(13/83) 15.7%	0.99
		*p*-value*	0.99	1	1	
		AG	(14/89) 15.7%	(61/89) 68.5%	(14/89) 15.7%	
		*p*-value *	0.99	0.99	1	
		GG	(5/38) 13.2%	(27/38) 71.1%	(6/38) 15.8%	
		*p*-value *	0.92	0.96	1	
	rs3184504	CC	(14/82) 17.1%	(56/82) 68.3%	(12/82) 14.6%	0.94
		*p*-value *	0.81	0.99	0.90	
		TC	(12/89) 13.5%	(61/89) 68.5%	(16/89) 18%	
		*p*-value *	0.86	1	0.81	
		TT	(6/41) 14.6%	(29/41) 70.7%	(6/41) 14.6%	
		*p*-value *	1	0.96	0.97	
*MTHFD1L*	rs491552	CC	(6/45) 13.3%	(34/45) 75.6%	(5/45) 11.1%	0.79
		*p*-value *	0.98	0.66	0.63	
		CG	(13/101) 12.9%	(71/101) 70.3%	(17/101) 16.8%	
		*p*-value *	0.86	1	0.90	
		GG	(10/58) 17.2%	(38/58) 65.5%	(10/58) 17.2%	
		*p*-value*	0.74	0.67	0.93	
	rs6922269	AA	(2/17) 11.8%	(14/17) 82.4%	(1/17) 5.9%	0.03
		*p*-value *	0.92	0.46	0.49	
		AG	(11/80) 13.8%	(48/80) 60%	(21/80) 26.2%	
		*p*-value *	0.91	0.09	0.01	
		GG	(19/115) 16.5%	(84/115) 73%	(12/115) 10.4%	
		*p*-value *	0.82	0.36	0.05	
	rs803422	AA	(2/24) 8.3%	(8/24) 75%	(4/24) 16.7%	0.58
		*p*-value *	0.62	0.79	1	
		GA	(15/75) 20%	(50/75) 66.7%	(10/75) 13.3%	
		*p*-value *	0.34	0.88	0.73	
		GG	(15/113) 13.3%	(78/113) 69%	(20/113) 17.7%	
		*p*-value *	0.73	1	0.78	
	rs803455	AA	(0/1) 0%	(1/1) 100%	(0/1) 0%	0.49
		*p*-value *	0.91	0.80	0.91	
		GA	(7/27) 25.9%	(17/27) 63%	(3/27) 11.1%	
		*p*-value *	0.25	0.76	0.79	
		GG	(25/183) 13.7%	(128/183) 69.9%	(30/183) 16.4%	
		*p*-value *	0.30	0.84	0.74	

* Chi-square test with *p*-value < 0.05 is considered significant. ^a^ Warfarin-sensitive group (required minimum warfarin dose < 21 mg/week). ^b^ Warfarin-moderate response group (required average warfarin dose between 21 and 49 mg/week). ^c^ Warfarin-resistance group (required high warfarin dose > 49 mg/week).

**Table 3:** Association of *SH2B3* and *MTHFD1L* SNPs with warfarin sensitivity during the stabilization phase of therapy of 139 cardiovascular patients treated with warfarin.

Gene	SNP ID	Genotype	Sensitive ^a^	Moderate ^b^	Resistance ^c^	Overall *p*-Value *
*SH2B3*	rs11065987	AA	(5/52) 9.6%	(36/52) 69.2%	(11/52) 21.2%	0.41
		*p*-value *	0.46	0.38	0.86	
		GA	(9/63) 14.3%	(37/63) 58.7%	(17/63) 27%	
		*p*-value *	1	0.79	0.72	
		GG	(6/24) 25%	(13/24) 54.2%	(5/24) 20.8%	
		*p*-value *	0.27	0.69	0.93	
	rs17696736	AA	(4/51) 7.8%	(36/51) 70.6%	(11/51) 21.6%	0.31
		*p*-value *	0.23	0.23	0.87	
		AG	(10/61) 16.4%	(35/61) 57.4%	(16/61) 26.2%	
		*p*-value *	0.87	0.70	0.87	
		GG	(6/25) 24%	(13/25) 52%	(6/25) 24%	
		*p*-value *	0.34	0.57	1	
	rs3184504	CC	(5/51) 9.8%	(36/51) 70.6%	(10/51) 19.6%	0.35
		*p*-value *	0.50	0.27	0.68	
		TC	(9/61) 14.8%	(34/61) 55.7%	(18/61) 29.5%	
		*p*-value *	1	0.42	0.37	
		TT	(6/27) 22.2%	(16/27) 59.3%	(5/27) 18.5%	
		*p*-value *	0.30	0.95	0.78	
*MTHFD1L*	rs491552	CC	(2/25) 8%	(15/25) 60%	(8/25) 32%	0.71
		*p*-value *	0.89	0.73	0.62	
		CG	(10/70) 14.3%	(46/70) 65.7%	(14/70) 20%	
		*p*-value *	0.98	0.82	0.45	
		GG	(5/36) 13.9%	(21/36) 58.3%	(10/36) 27.8%	
		*p*-value *	0.96	0.62	0.86	
	rs6922269	AA	(2/11) 18.2%	(7/11) 63.6%	(2/11) 18.2%	0.05
		*p*-value *	0.93	0.99	0.90	
		AG	(7/57) 12.3%	(29/57) 50.9%	(21/57) 36.8%	
		*p*-value *	0.84	0.08	0.01	
		GG	(11/71) 15.5%	(50/71) 70.4%	(10/71) 14.1%	
		*p*-value *	0.93	0.11	0.02	
	rs803422	AA	(2/14) 14.3%	(7/14) 50%	(5/14) 35.7%	0.64
		*p*-value *	1	0.63	0.54	
		GA	(9/52) 17.3%	(30/52) 57.7%	(13/52) 25%	
		*p*-value *	0.75	0.74	0.97	
		GG	(9/73) 12.3%	(49/73) 67.1%	(15/73) 20.5%	
		*p*-value *	0.77	0.41	0.65	
	rs803455	GA	(4/20) 20%	(12/20) 60%	(4/20) 20%	0.74
		*p*-value *	0.75	0.98	0.94	
		GG	(16/118) 13.6%	(74/118) 62.7%	(28/118) 23.7%	
		*p*-value *	0.75	0.98	0.94	

* Chi-square test with *p*-value < 0.05 is considered significant. ^a^ Warfarin-sensitive group (required minimum warfarin dose < 21 mg/week). ^b^ Warfarin-moderate response group (required average warfarin dose between 21 and 49 mg/week). ^c^ Warfarin-resistance group (required high warfarin dose > 49 mg/week).

**Table 4:** Association of *SH2B3* and *MTHFD1L* SNPs with the variability on warfarin required doses.

Gene	SNP ID	Genotype	Initiation Dose	Overall *p*-Value *	Maintenance Dose	Overall *p*-Value *
*SH2B3*		AA	35.66 (12.87)		37.31 (14.39)	
	rs11065987	GA	37.32 (14.74)	0.1	40.00 (19.24)	0.69
		GG	45.32 (46.11)		37.48 (21.18)	
		AA	36.03 (12.70)		37.90 (14.11)	
	rs17696736	AG	36.74 (14.78)	0.09	38.42 (17.37)	0.98
		GG	45.36 (45.48)		38.00 (20.90)	
		CC	35.70 (12.74)		36.61 (13.60)	
	rs3184504	TC	37.92 (14.86)	0.17	41.07 (19.83)	0.35
		TT	43.74 (44.11)		36.57 (20.12)	
*MTHFD1L*		CC	41.17 (40.85)		39.06 (14.54)	
	rs491552	CG	38.13 (15.48)	0.62	39.76 (19.65)	0.88
		GG	36.65 (14.47)		37.84 (17.42)	
		AA	34.64 (09.87)		35.25 (13.99)	
	rs6922269	AG	43.91 (33.84)	0.02	44.52 (22.01)	0.004
		GG	34.49 (11.81)		34.28 (12.84)	
		AA	38.53 (13.39)		40.86 (18.38)	
	rs803422	GA	36.58 (15.18)	0.79	38.63 (19.85)	0.87
		GG	38.94 (28.49)		38.07 (16.45)	
		AA	34.80 (……)			
	rs803455	GA	33.11 (14.99)	0.49	35.22 (15.28)	0.39
		GG	38.60 (24.01)		38.85 (18.11)	

* One-way ANOVA test with *p*-value < 0.05 is considered significant; mean standard deviation in roundbrackets.

**Table 5:** Association of *SH2B3* and *MTHFD1L* SNPs with warfarin responsiveness during the initiation phase of therapy of 212 cardiovascular patients treated with warfarin.

Gene	SNP ID	Genotype	Poor ^a^	Good ^b^	Ultra ^c^	Overall *p*-Value *
*SH2B3*	rs11065987	AA	(17/84) 20.2%	(62/84) 73.8%	(5/84) 6%	0.95
		*p*-value *	0.86	0.77	0.92	
		GA	(15/91) 16.5%	(72/91) 79.1%	(4/91) 4.4%	
		*p*-value *	0.82	0.72	0.90	
		GG	(7/37) 18.9%	(28/37) 75.7%	(2/37) 5.4%	
		*p*-value *	1	1	1	
	rs17696736	AA	(17/83) 20.5%	(62/83) 74.7%	(4/83) 4.8%	0.98
		*p*-value *	0.85	0.92	0.98	
		AG	(15/89) 16.9%	(69/89) 77.5%	(5/89) 5.6%	
		*p*-value *	0.85	0.93	0.98	
		GG	(7/38) 18.4%	(29/38) 76.3%	(2/38) 5.3%	
		*p*-value *	1	1	1	
	rs3184504	CC	(17/82) 20.7%	(60/82) 73.2%	(5/82) 6.1%	0.89
		*p* -value *	0.78	0.69	0.98	
		TC	(14/89) 15.7%	(71/89) 79.8%	(4/89) 4.5%	
		*p*-value *	0.68	0.62	0.99	
		TT	(8/41) 19.5%	(31/41) 75.6%	(2/41) 4.9%	
		*p*-value *	0.90	0.93	1	
*MTHFD1L*	rs491552	CC	(11/45) 24.4%	(34/45) 75.6%	(0/45) 0%	0.07
		*p*-value *	0.59	1	0.19	
		CG	(18/101) 17.8%	(79/101) 78.2%	(4/101) 4%	
		*p*-value *	0.90	0.67	0.67	
		GG	(10/58) 17.2%	(41/58) 70.7%	(7/58) 12.1%	
		*p*-value *	0.91	0.6	0.03	
	rs6922269	AA	(4/17) 23.5%	(11/17) 64.7%	(2/17) 11.8%	0.56
		*p*-value *	0.85	0.49	0.44	
		AG	(15/80) 18.8%	(60/80) 75%	(5/80) 6.3%	
		*p*-value *	1	0.93	0.87	
		GG	(20/115) 17.4%	(91/115) 79.1%	(4/115) 3.5%	
		*p*-value *	0.92	0.6	0.47	
	rs803422	AA	(4/24) 16.7%	(20/24) 83.3%	(0/24) 0%	0.55
		*p*-value *	0.98	0.70	0.48	
		GA	(11/75) 14.7%	(60/75) 80%	(4/75) 5.3%	
		*p*-value *	0.58	0.66	1	
		GG	(24/113) 21.2%	(82/113) 72.6%	(7/113) 6.2%	
		*p*-value *	0.73	1	0.78	
	rs803455	AA	(0/1) 0%	(1/1) 100%	(0/1) 0%	0.64
		*p*-value *	0.89	0.86	0.97	
		GA	(4/27) 14.8%	(23/27) 85.2%	(0/27) 0%	
		*p*-value *	0.87	0.51	0.43	
		GG	(35/183) 19.1%	(137/183) 74.9%	(11/183) 6%	
		*p*-value *	0.83	0.45	0.41	

* Chi-square test with *p*-value < 0.05 is considered significant. ^a^ Poor responders (international normalized ratio (INR) value below target). ^b^ Good responders who have an INR in the target range (therapeutic range). ^c^ Ultra-responders (INR over target).

**Table 6:** Association of *SH2B3* and *MTHFD1L* SNPs with warfarin responsiveness during the stabilization phase of therapy of 139 cardiovascular patients treated with warfarin.

Gene	SNP ID	Genotype	Poor ^a^	Good ^b^	Ultra ^c^	Overall *p*-Value *
*SH2B3*	rs11065987	AA	(4/52) 7.7%	(44/52) 84.6%	(4/52) 7.7%	0.56
		*p*-value *	0.90	0.40	0.32	
		GA	(4/63) 6.3%	(58/63) 92.1%	(1/63) 1.6%	
		*p*-value *	1	0.61	0.35	
		GG	(1/24) 4.2%	(22/24) 91.7%	(1/24) 4.2%	
		*p*-value **	0.88	0.91	1	
	rs17696736	AA	(4/51) 7.8%	(44/51) 86.3%	(3/51) 5.9%	0.93
		*p*-value *	0.90	0.73	0.80	
		AG	(4/61) 6.6%	(55/61) 90.2%	(2/61) 3.3%	
		*p*-value *	1	0.93	0.85	
		GG	(1/25) 4%	(23/25) 92%	(1/25) 4%	
		*p*-value *	0.85	0.87	1	
	rs3184504	CC	(4/51) 7.8%	(43/51) 84.3%	(4/51) 7.8%	0.53
		*p*-value *	0.88	0.37	0.30	
		TC	(4/61) 6.6%	(56/61) 91.8%	(1/61) 1.6%	
		*p*-value *	1	0.68	0.39	
		TT	(1/27) 3.7%	(25/27) 92.6%	(1/27) 3.7%	
		*p*-value *	0.81	0.82	0.99	
*MTHFD1L*	rs491552	CC	(2/25) 8%	(23/25) 92%	(0/25) 0%	0.26
		*p*-value *	0.97	0.84	0.48	
		CG	(5/70) 7.1%	(63/70) 90%	(2/70) 2.9%	
		*p*-value *	0.99	0.86	0.60	
		GG	(2/36) 5.6%	(30/36) 83.3%	(4/36) 11.1%	
		*p*-value *	0.94	0.51	0.09	
	rs6922269	AA	(3/11) 27.3%	(6/11) 54.5%	(2/11) 18.2%	0.30
		*p*-value *	0.01	<0.0001	0.06	
		AG	(4/57) 7%	(51/57) 89.5%	(2/57) 3.5%	
		*p*-value *	0.98	1	0.93	
		GG	(2/71) 2.8%	(67/71) 94.4%	(2/71) 2.8%	
		*p*-value *	0.2	0.13	0.67	
	rs803422	AA	(0/14) 0%	(14/14) 100%	(0/14) 0%	0.18
		*p*-value *	0.58	0.39	0.70	
		GA	(1/52) 1.9%	(49/52) 94.2%	(2/52) 3.8%	
		*p*-value *	0.24	0.34	0.98	
		GG	(8/73) 11%	(61/73) 83.6%	(4/73) 5.4%	
		*p*-value *	0.08	0.08	0.78	
	rs803455	GA	(0/20) 0%	(20/20) 100%	(0/20) 0%	0.24
		*p*-value *	0.44	0.24	0.59	
		GG	(9/118) 7.6%	(103/118) 87.3%	(6/118) 5.1%	
		P-value*	0.44	0.24	0.59	

* Chi-square test with *p*-value < 0.05 is considered significant. ^a^ Poor responders (INR value below target). ^b^ Good responders who have an INR value in the target range (therapeutic range). ^c^ Ultra-responders (INR over target).

**Table 7:** Association of *SH2B3* and *MTHFD1L* SNPs with the INR treatment outcomes.

Gene	SNP ID	Genotype	Initiation INR	Overall *p*-Value *	Maintenance INR	Overall *p*-Value *
*SH2B3*		AA	2.41 (0.75)		2.68 (0.43)	
	rs11065987	GA	2.51 (0.79)	0.70	2.68 (0.37)	0.80
		GG	2.45 (0.79)		2.74 (0.35)	
		AA	2.39 (0.75)		2.69 (0.42)	
	rs17696736	AG	2.53 (0.80)	0.53	2.68 (0.38)	0.86
		GG	2.44 (0.78)		2.73 (0.34)	
		CC	2.41 (0.76)		2.68 (0.43)	
	rs3184504	TC	2.50 (0.77)	0.72	2.68 (0.38)	0.75
		TT	2.46 (0.81)		2.74 (0.33)	
*MTHFD1L*		CC	2.33 (0.75)		2.67 (0.39)	
	rs491552	CG	2.40 (0.69)	0.15	2.61 (0.37)	0.02
		GG	2.61 (0.93)		2.83 (0.40)	
		AA	2.30 (0.67)		2.66 (0.49)	
	rs6922269	AG	2.55 (0.82)	0.73	2.66 (0.42)	0.65
		GG	2.42 (0.76)		2.72 (0.35)	
		AA	2.46 (0.97)		2.64 (0.37)	
	rs803422	GA	2.47 (0.50)	0.62	2.73 (0.41)	0.68
		GG	1.70 (……)		2.68 (0.38)	
		AA	1.90 (……)			
	rs803455	GA	2.48 (0.66)	0.76	2.85 (0.31)	0.05
		GG	2.46 (0.79)		2.67 (0.40)	

* One-way ANOVA test with *p*-value < 0.05 is considered significant; mean standard deviation in round brackets.

## Supplementary Materials

The supplemental materials for this project are available online at https://www.mdpi.com/2075-4426/10/3/117/s1. Table S1: Genes and SNPs Characteristics. Table S2: List of SNPs, their minor allele frequencies, and HWE p-values. Table S3: The distributions of *SH2B3* and *MTHFD1L* haplotypes and 211 Cardiovascular patients in compare to 213 healthy controls. Table S4: The distributions of *SH2B3* and *MTHFD1L* haplotypes among 212 warfarin sensitive patients. Table S5: Post Hoc Tests for theAssociation of *SH2B3* and *MTHFD1L* SNPs with variability on warfarin required doses. Table S6: The distributions of *SH2B3* and *MTHFD1L* haplotypes among 212 warfarin responsiveness patients. Table S7: Post Hoc Tests for theAssociation of *SH2B3* and *MTHFD1L* SNPs with INR Treatment Outcome.
