# Supplementary material for: Influence of SH2B3, MTHFD1L, GGCX, and ITGB3 Gene Polymorphisms on theVariability on Warfarin Dosage Requirements and Susceptibility to CVD in the Jordanian Population
Source: J Pers Med. 2020 Sep 9;10(3):117. doi: 10.3390/jpm10030117 (PMC7564501; doi:10.3390/jpm10030117)
Supplement: Supplementary file 1 [file jpm-10-00117-s001.pdf]

Table S1. Genes and SNPs characteristics.

Gene	SNP ID	ChrPosition[a]	SNP	SNP Type
	rs11065987	12:111634620	A/G	N/A[b]
SH2B3	rs17696736	12:112049014	A/G	Intron Variant
	rs3184504	12:111446804	T/C	Missense Variant
	rs491552	6:150989919	C/G	Intron Variant
MTHFD1L	rs6922269	6:150931849	G/A	Intron Variant
	rs803422	6:150894458	A/G	Intron Variant
	rs803455	6:150926303	A/G	Intron Variant
GGCX	rs28928872	2:85551919	C/G	Missense Variant
ITGB3	rs398122372	17:47307581	G/A<br>G/C	Missense Variant
	rs398122374	17:47307567	T/A<br>T/C<br>T/G	Missense Variant

[a.] Chromosome positions are based on NCBI Human Genome Assembly Build.
[b.]N/A: Not Avilable.

Table S2. List of SNPs, their minor allele frequencies, and HWE p-values.

Gene	SNP ID	MA[a]	Patients MAF[b]	Controls MAF[b]	HWE[c] *P*-value
	rs11065987	G	0.39	0.37	**0.03**
SH2B3	rs17696736	G	0.39	0.36	0.05
	rs3184504	T	0.4	0.37	**0.03**
	rs491552	C	0.47	0.46	0.08
MTHFD1L	rs6922269	A	0.27	0.25	0.62
	rs803422	A	0.29	0.23	0.05
	rs803455	A	0.07	0.11	0.36

[a.] MA: Minor allele.
[b.] MAF: Minor allele frequency.
[c.] HWE: Hardy-Weinberg equilibrium.

Table S3. The distributions of *SH2B3 and MTHFD1L* haplotypes and 211 cardiovascular patients in compare to 213 healthy controls.

Gene	Haplotypes	Patients (%)	Controls (%)	Odds ratio (95% CI)	*P*-value*
SH2B3	AAC	0.59	0.62	1.00	---
	GGT	0.39	0.35	1.13 (0.86 - 1.48)	0.37
	AAT	0.01	0.007	2.05 (0.50 - 8.33)	0.32
MTHFD1L	GGGG	0.23	0.30	1.00	---
	CGGG	0.21	0.19	1.26 (0.78 - 2.04)	0.35
	CAGG	0.11	0.09	1.35 (0.68 - 2.68)	0.4
	GAGG	0.11	0.09	1.39 (0.76 - 2.53)	0.29
	GGAG	0.13	0.07	1.98 (1.04 - 3.78)	**0.04**
	CGAG	0.01	0.09	1.41 (0.77 - 2.56)	0.26
	CGGA	0.03	0.04	1.16 (0.46 - 2.91)	0.75
	GAAG	0.04	0.02	3.50 (0.79 - 15.53)	0.1
	GGGA	0.03	0.03	0.71 (0.12 - 4.28)	0.71
	CAAG	0.01	0.03	0.42 (0.05 - 3.33)	0.41
	GGAA	0.0	0.02	0.44 (0.01 - 12.88)	0.63

*Chi–Square Test with $p<0.05$ is considered significant.

Table S4. The distributions of *SH2B3 and MTHFD1L* haplotypes among 212 warfarin sensitive patients.

Gene	Haplotypes	Frequency (%)	Odds ratio (95% CI)	*P*-value*
SH2B3	AAC	0.47	0.00	---
	GGT	0.26	0.02 (-0.11 - 0.14)	0.77
	AGC	0.13	0.04 (-0.14 - 0.21)	0.67
	GAT	0.13	0.06 (-0.12 - 0.24)	0.52
	AAT	0.01	-0.01 (-0.47 - 0.45)	0.98
MTHFD1L	GGGG	0.26	0.00	---
	CGGG	0.17	-0.15 (-0.36 - 0.06)	0.16
	CAGG	0.13	0.14 (-0.09 - 0.36)	0.23
	GGAG	0.13	-0.09 (-0.33 - 0.16)	0.5
	CGAG	0.10	-0.09 (-0.3 - 0.12)	0.4
	GAGG	0.1	-0.18 (-0.47 - 0.11)	0.22
	CGGA	0.03	-0.22 (-0.62 - 0.17)	0.27
	CAAG	0.03	-0.05 (-0.49 - 0.38)	0.81
	GAAG	0.02	0.05 (-0.53 - 0.62)	0.87
	GGGA	0.02	-0.2 (-0.68 - 0.27)	0.4
	CGAA	0.01	-0.13 (-0.65 - 0.39)	0.62

*Chi–Square Test with p<0.05 is considered significant.

Table S5. Post Hoc tests for the association of *SH2B3* and *MTHFD1L* SNPs with variability on warfarin required doses.

Gene	SNP ID	Genotype		Initiation Dose *P*-value*	Maintenance Dose *P*-value*
SH2B3	rs11065987	AA	GA	0.88	0.70
			GG	0.09	1
		GA	AA	0.88	0.70
			GG	0.18	0.83
		GG	AA	0.09	1
			GA	0.18	0.83
	rs17696736	AA	AG	0.98	0.99
			GG	0.1	1
		AG	AA	0.98	0.99
			GG	0.13	0.99
		GG	AA	0.1	1
			AG	0.13	0.99
	rs3184504	CC	TC	0.75	0.39
			TT	0.14	1
		TC	CC	0.75	0.39
			TT	0.37	0.52
		TT	TC	0.14	1
			TT	0.37	0.52
MTHFD1L	rs491552	CC	CG	0.75	0.99
			GG	0.60	0.96
		CG	CC	0.75	0.99
			GG	0.92	0.86
		GG	CC	0.60	0.96
			CG	0.92	0.86
	rs6922269	AA	AG	0.28	0.24
			GG	1	0.98
		AG	AA	0.28	0.24
			GG	**0.01**	**0.003**
		GG	AA	1	0.98
			AG	**0.01**	**0.003**
	rs803422	AA	GA	0.93	0.91
			GG	1	0.86
		GA	AA	0.93	0.91
			GG	0.77	0.98
		GG	AA	1	0.86
			GA	0.77	0.98

*Post-HockMultiple comparisons Test with p<0.05 is considered significant. Compare means of the initiation and maintenance dose among all genotypes.
Post hoc tests are not performed for rs803455because at least one group has fewer than two cases.

Table S6. The distributions of *SH2B3 and MTHFD1L* haplotypes among 212 warfarin responsiveness patients.

Gene	Haplotypes	Frequency (%)	Odds ratio (95% CI)	*P*-value*
SH2B3	AAC	0.47	0.00	---
	GGT	0.26	0 (-0.1 - 0.1)	0.99
	AGC	0.13	**0.22 (0.08 - 0.36)**	**0.002**
	GAT	0.13	**0.23 (0.09 - 0.38)**	**0.002**
MTHFD1L	GGGG	0.26	0.00	---
	CGGG	0.19	-0.14 (-0.29 - 0.02)	0.085
	GGAG	0.12	-0.09 (-0.26 - 0.07)	0.28
	GAGG	0.12	-0.02 (-0.23 - 0.18)	0.83
	CAGG	0.10	-0.05 (-0.23 - 0.14)	0.61
	CGAG	0.10	-0.18 (-0.38 - 0.02)	0.08
	CGGA	0.03	0.09 (-0.16 - 0.35)	0.48
	CAAG	0.03	-0.05 (-0.35 - 0.24)	0.73
	GAAG	0.02	0.1 (-0.28 - 0.47)	0.61
	GGGA	0.02	0.08 (-0.27 - 0.42)	0.67
	CGAA	0.01	-0.38 (-0.92 - 0.16)	0.17

*Chi–Square Test with $p<0.05$ is considered significant.

Table S7. Post Hoc Tests for theAssociation of *SH2B3* and *MTHFD1L* SNPs with INR Treatment Outcome.

Gene	SNP ID	Genotype		Initiation INR *P*-value*	MaintenanceINR *P*-value*
SH2B3	rs11065987	AA	GA	0.68	1
			GG	0.96	0.82
		GA	AA	0.68	1
			GG	0.92	0.81
		GG	AA	0.96	0.82
			GA	0.92	0.81
	rs17696736	AA	AG	0.50	1
			GG	0.94	0.89
		AG	AA	0.50	1
			GG	0.85	0.85
		GG	AA	0.94	0.89
			AG	0.85	0.85
	rs3184504	CC	TC	0.70	1
			TT	0.94	0.76
		TC	CC	0.70	1
			TT	0.95	0.77
		TT	CC	0.94	0.76
			TC	0.95	0.77
MTHFD1L	rs491552	CC	CG	0.86	0.75
			GG	0.17	0.24
		CG	CC	0.86	0.75
			GG	0.24	**0.01**
		GG	CC	0.17	0.24
			CG	0.24	**0.01**
	rs6922269	AA	AG	0.85	1
			GG	0.72	0.88
		AG	AA	0.85	1
			GG	0.92	0.65
		GG	AA	0.72	0.88
			AG	0.92	0.65
	rs803422	AA	GA	0/34	0.75
			GG	0.75	0.95
		GA	AA	0.34	0.75
			GG	0.50	0.75
		GG	AA	0.75	0.95
			GA	0.50	0.75

*Post-HockMultiple comparisons Test with p<0.05 is considered significant. Compare initiation and maintenance dose among all genotypes.
Post hoc tests are not performed for rs803455because at least one group has fewer than two cases.
